# On the Eyes of Male Coffee Berry Borers as Rudimentary Organs

**DOI:** 10.1371/journal.pone.0085860

**Published:** 2014-01-20

**Authors:** Fernando E. Vega, Ann Simpkins, Gary Bauchan, Francisco Infante, Matthew Kramer, Michael F. Land

**Affiliations:** 1 Sustainable Perennial Crops Laboratory, United States Department of Agriculture, Agricultural Research Service, Beltsville, Maryland, United States of America; 2 Electron and Confocal Microscopy Unit, United States Department of Agriculture, Agricultural Research Service, Beltsville, Maryland, United States of America; 3 El Colegio de la Frontera Sur, Tapachula, Chiapas, México; 4 Biometrical Consulting Service, United States Department of Agriculture, Agricultural Research Service, Beltsville, Maryland, United States of America; 5 School of Life Sciences, University of Sussex, Brighton, United Kingdom; University of Tennessee, United States of America

## Abstract

The coffee berry borer, *Hypothenemus hampei*, is the most damaging insect pest of coffee worldwide. Like males in other species in the genus, male coffee berry borers have a lower number of facets in the compound eyes than females. The rudimentary eyes in male coffee berry borers could be an evolutionary response to their cryptic life habit, whereby they are born inside a coffee berry and never leave the berry. The main objective of the study was to determine if the differences in the number of facets translates into differences in visual acuity. We used low-temperature scanning electron microscopy to visualize and quantify the number of facets in the compound eyes. There was a significantly lower (*p*<0.0001) number of facets in males (19.1±4.10) than in females (127.5±3.88). To assess visual acuity, we conducted optomotor response experiments, which indicate that females respond to movement, while males did not respond under the conditions tested. The coffee berry borer is an example of an insect whereby disuse of an organ has led to a rudimentary compound eye. This is the first study that has experimentally tested responses to movement in bark beetles.

## Introduction

*I believe that disuse … has led in successive generations to the gradual reduction of various organs, until they have become rudimentary, - as in the case of the eyes of animals inhabiting dark caverns, and of the wings of birds inhabiting oceanic islands, which have seldom been forced to take flight, and have ultimately lost the power of flying. Again, an organ useful under certain conditions, might become injurious under others, … and in this case natural selection would continue slowly to reduce the organ, until it was rendered harmless and rudimentary.*

Charles Darwin, *On the Origin of Species* [1].

The coffee berry borer, *Hypothenemus hampei* (Ferrari) (Coleoptera: Curculionidae, Scolytinae), is the most damaging insect pest of coffee worldwide. Yearly losses caused by this insect in Brazil alone have been estimated at US$285–315 million [2]. Adult females bore a hole in or near the apex of the coffee berry (botanically known as the disc), and oviposit within galleries in the endosperm. There is a skewed sex ratio favoring females followed by sibling mating, with males being smaller (1.0–1.3 mm) than females (1.6–1.9 mm), flightless, and never leaving the berry [3]–[5].

During scanning electron microscopy-based research aimed at visualizing various morphological features in the coffee berry borer, we observed dramatic differences in the number of facets in the compound eyes of female and male insects. The compound eyes of insects consist of a series of individual photoreceptor units called ommatidia comprising the lens, receptors, and associated structures [6]. Each ommatidium corresponds to a dome or facet, which is visible on the surface of the compound eye.

A few papers have reported, mostly superficially, on the vision and ommatidia of bark beetles (Table 1), with the exception of four papers that provide more detailed information [7]–[10]. Most of the papers listed in Table 1 do not provide sample sizes or statistical analyses and to our knowledge, there are no reports in the literature dealing with bark beetle responses to movement.

**Table 1:** Number of facets reported in the literature for various bark beetles. Sex, sample size, and statistical analyses presented when given in original paper.

Scolytinae	# facets	Reference
*Dendroctonus pseudotsugae* 1	302	[7]
*D. rufipennis* 1	272	[7]
*D. valens* – mixed sexes	372	[7]
*Dryocoetes autographus* – mixed sexes	138	[7]
*Hylastes nigrinus* – mixed sexes	178	[7]
*Trypodendron lineatum* – females	173	[7]
*Xyleborus ferrugineus* – females	72–105	[8]
*X. ferrugineus* – males	19–33	[9]
*Coccotrypes dactyliperda* – females	75–104	[10]
*C. dactyliperda* – cavernicolous females	65	[10]
*Chaetoptelius vestitus*	ca. 250	[11]
*Dendroctonus*	ca. 400	[12]
*Hypothenemus hampei* – females	115	[13]
*H. obscurus* – females	150	[13]
*Ips typographus*	215	[14]
*Pityogenes chalcographus*	110	[14]
*Scolytus laevis*	235	[14]
*Tomicus piniperda*	215	[14]

^1^ No significant differences based on sex.

Our goal was to determine whether there are differences not only in the number of facets between male and female coffee berry borers, but also on their ability to respond to movement. We decided to use movement rather than color because males spend their entire life cycle inside the coffee berry, don’t see light, and have no visually guided behavior, in contrast to females, which need to fly and find berries. Motion detection is crucial for maintaining orientation during flight [15] and is straightforward to test, whereas testing for color or shape generally requires training (as in bees) or strong, and in the case for the coffee berry borer, unknown, innate preferences.

A simple method used to assess motion vision in insects is to place the subject in an optomotor response apparatus consisting of a rotating striped drum [15]. We used low temperature-scanning electron microscopy to quantify differences in the number of facets and conducted experiments to determine the optomotor response in male and female coffee berry borers.

## Materials and Methods

### Insects

Coffee berry borers were reared in 20 ml clear glass scintillation vials containing an artificial diet based on ground green coffee and several other ingredients [16]. The vials containing the insects were kept in the dark in a growth chamber set at 28°C. The insects used in the bioassays were 1–3 months old and all insects were considered to be mated.

### Low Temperature-Scanning Electron Microscopy (LT-SEM)

LT-SEM was used to observe, record, and subsequently count the number of facets in left and right eyes for male and female coffee berry borers. Due to the high number of facets in females, it was necessary to excise the antenna, which obstructs some of the facets. Removal of antennae was not essential for males due to the low number of facets. We used LT-SEM because it does not require any fixation, critical point drying, or other preparations with the exception to freezing in liquid nitrogen prior to insertion into the SEM, thus eliminating any artifacts and possible distortion of specimens.

Specimens were secured to 15×30 cm copper plates with a thin layer of Tissue Freezing Medium (Triangle Biomedical Sciences, Durham, NC), which acted as the cryo-adhesive upon freezing. The specimens were frozen conductively in a Styrofoam box by placing the plates on the surface of a pre-cooled (−196°C) brass bar whose lower half was submerged in liquid nitrogen. After 20–30 seconds, the holders containing the frozen samples were transferred to a Quorum PP2000 cryo-preparation chamber (Quorum Technologies, East Sussex, UK) attached to an S-4700 field emission scanning electron microscope (Hitachi High Technologies America, Inc., Pleasanton, CA). The specimens were etched inside the cryotransfer system to remove any surface contamination (e.g., condensed water vapor) by raising the temperature of the stage to −90°C for 10–15 minutes. Following etching, the temperature inside the chamber was lowered below −130°C, and the specimens were coated with a 10 nm layer of platinum using a magnetron sputter head equipped with a platinum target. The specimens were transferred to a pre-cooled (−130°C) cryostage in the SEM for observation. An accelerating voltage of 5 kV was used to view the specimens. Images were captured using a 4 pi Analysis System (Durham, NC).

### Optomotor Response Apparatus

**Figure 1:**
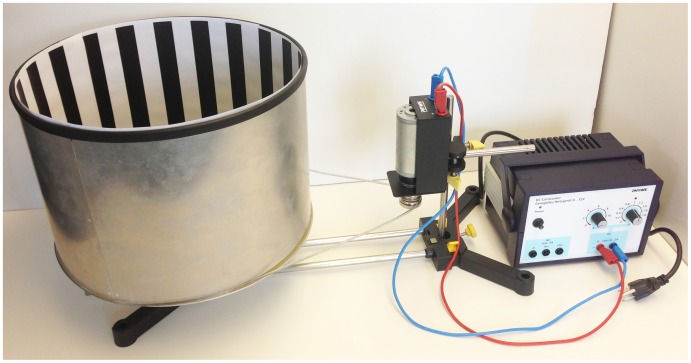
Optomotor response apparatus. Apparatus used to assess coffee berry borer optomotor response. Striped pattern consisted of 2.54(20 stripes of each color).

An apparatus useful in measuring optomotor response in insects was acquired from PHYWE Systeme GmbH & Co. KG (cat. no. P4070100; Göttingen, Germany; Figure 1). The apparatus consists of a drum with a rotating striped pattern encircling a stationary (31 cm diameter) arena. The drum is connected to a 12 V motor and power supply used to adjust revolutions per minute (rpm). We conducted preliminary trials at different rpm using the 0.61 cm wide and 17.8 cm long vertical black and white stripes supplied with the rotating drum (180 stripes of each color). None of the trials resulted in discernible female coffee berry borer responses. We replaced the original striped pattern with 2.54 cm wide vertical black and white stripes (20 stripes of each color; 18° per stripe), which resulted in discernible female optomotor responses when a drum speed of 13 rpm was used. This gave a pattern speed of 78° per second as seen by the insect. This set up was used in all subsequent tests, which were conducted in a walk-in growth chamber (Environmental Growth Chambers, Model GC72, Chagrin Falls, OH) set at 25°C, 60% relative humidity, and with 3.55 lux inside the drum. Each morning the experiments were to be conducted (8∶00 - 10∶00 AM), insects were removed from artificial diet [16] and placed in empty 20 ml clear glass scintillation vials; these were kept in the dark inside a box in the walk-in growth chamber until used in the bioassays. For each test, which ran for 5 minutes, an individual insect was placed in the center of the arena. We then counted the number of turns each beetle made to the left (following drum movement) and to the right as it walked within the arena.

### Statistical Analysis

#### Facets and interommatidial angles

Only one eye (left or right) could be sampled on each insect due to the insect being secured on its side to the copper plate with Tissue Freezing Medium. The sample size for male left and right eyes was 11 and 13, respectively. For females, 17 left eyes and 13 right eyes were sampled. Means and mean separations (one-way ANOVA) were calculated using SAS JMP [17].

Interommatidial angles (Δ*Φ*) can be used as a measurement of visual acuity, since each light-dark line pair in a grating requires two ommatidia to detect it, giving the finest resolvable grating an angular period of twice the inter-ommatidial angle (2Δ*Φ*). Δ*Φ* can be calculated approximately from facet numbers using the following formula: Δ*Φ* = (23,818/*n*)^½^, where 23,818 is the number of hexagons spaced 1° apart that will cover a hemisphere, and *n* is the number of facets Δ*Φ* degrees apart that will cover the same area [18].

#### Optomotor response

We individually tested female (n = 46 with drum on and n = 52 with drum off) and male beetles (n = 15 with drum on and n = 25 with drum off) once in the first set of tests, and tested males twice (n = 20) in the second set of tests consisting of paired tests with the drum on or off, with drum condition randomized.

If the drum was turned off, one would expect the number of left and right turns to be similar. If there was an effect of the rotating drum on the beetle, one would expect there to be more left than right turns (beetles following a counterclockwise rotating drum). Thus a test against a 50∶50 left:right distribution is appropriate. However, individual beetles could also have a left:right turning bias when the drum was off, so a more sensitive test is to ask whether the proportions of left to right turns for an individual beetle changed when the drum was moving versus stationary (motor off). This can be easily done with paired design, where each beetle is tested twice, once in the drum off condition and once in the drum on condition.

For each beetle in both sets of tests, a chi-squared test (2×2 contingency table) was conducted. In the first set of tests, this was against a 50∶50 left:right ratio. We then used these results to do another chi-squared test (also 2×2 contingency table), where we compared the number of significant tests in the drum-off to the drum-on conditions, i.e., each beetle contributed one observation to the counts in one of the four cells in the table. In the second set of (paired) tests (using male beetles only), for each beetle we tested whether the left:right ratio for drum-off differed from the drum-on condition.

We report on turning biases (drum-off condition), whether the left:right ratio changed when the drum was turned on (indicating that beetles perceived the moving drum), and whether males differed from females, as life style and morphology (see Introduction) suggest.

## Results

### Facets

**Figure 2:**
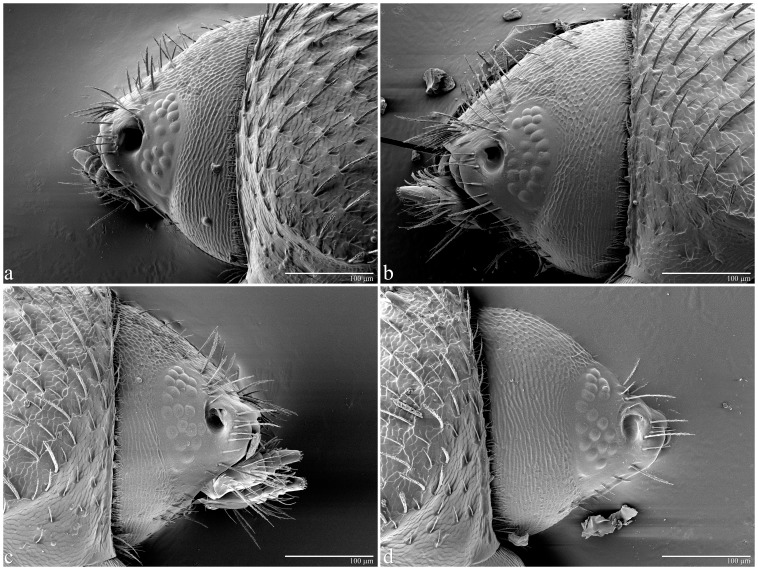
Coffee berry borer male eyes. LT-SEM of coffee berry borer male eyes to show variability in number of facets for left (a = 16; b = 18) and right (c = 19; d = 17) eyes. Note that the antennae have been removed.

**Figure 3:**
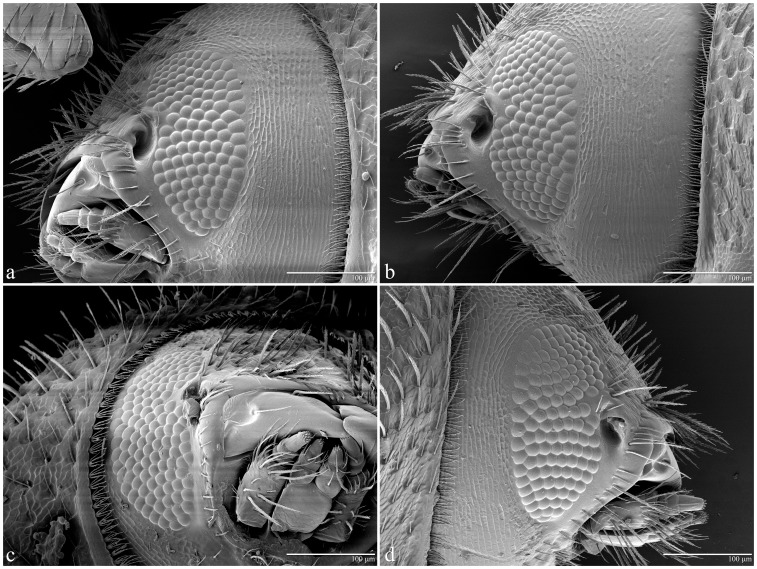
Coffee berry borer female eyes. LT-SEM of coffee berry borer female eyes to show variability in number of facets for left (a = 124; b = 128) and right (c = 131; d = 124) eyes. Note that the antennae have been removed.

The mean (± S.D.) number of facets in males’ left and right eyes was 17.8±3.95 (n = 11) and 20.2±4.04 (n = 13), respectively (Table 2). For females, the mean number was 127.3±4.07 (n = 17) and 127.7±3.77 (n = 13) for left and right eyes, respectively (Table 2). There were no significant differences in number of facets within sex (F_1,22_ = 2.17, *p* = 0.16, males; F_1,28_ = 0.75, *p* = 0.79, females; Table 2) but there was a significant difference (F_1,22_ = 2.17, *p*<0.0001) in the overall number of facets in males (19.1±4.10) vs females (127.5±3.88). Insects exhibited a wide range of facets: 12–27 in males and 122–136 in females (Table 2), with females exhibiting 5–10 times as many facets as males. Note that despite males having far fewer facets, the variability in the number of facets was about the same for males and females (S.D. about 4 for both groups). A sample of the variable number of facets is shown in Figure 2 for males and in Figure 3 for females. The estimated interommatidial angle (Δ*Φ*) for males was 35.3° [(23,818/19.1)^½^] and 13.6° [(23,818/127.6)^½^] for females.

**Table 2:** Means, range, and mean separation for number of facets in left and right eyes of male and female coffee berry borers.

	# Facets in Males		# Facets in Females	
	Left(n = 11)	Right(n = 13)	Left(n = 17)	Right(n = 13)
Range	13–24	12–27	122–135	123–136
Mean ± S.D.*	17.8±3.95	20.2±4.04	127.3±4.07	127.7±3.77
Mean ± S.D.**	19.1±4.10		127.5±3.88	

Non-significant differences within sex: males, *p* = 0.16; females, *p* = 0.79.

Significant difference, *p*<0.0001.

### Optomotor Response

**Figure 4:**
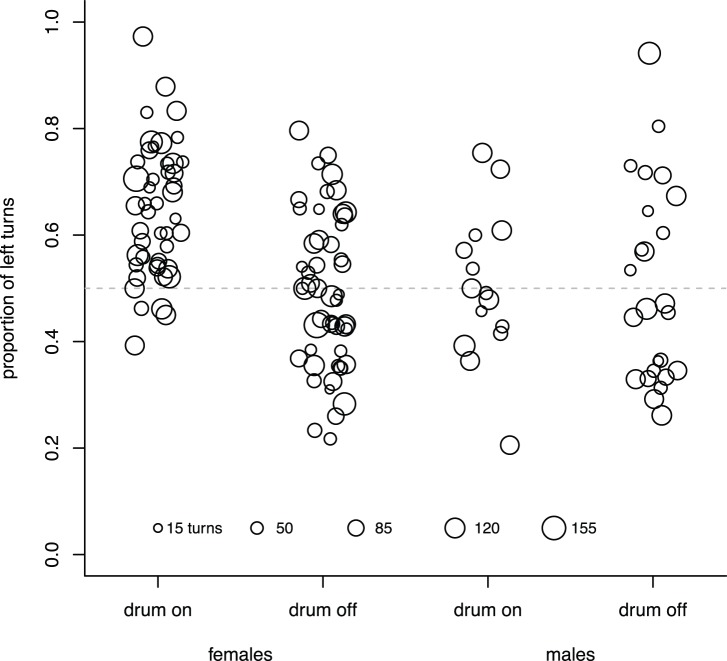
Proportion of left turns with drum-off and drum-on. The proportion of left turns given for each group of beetles (factors are sex and whether the drum was on or off) for the first set of tests. The total number of turns each beetle made is depicted by the size of the bubble, examples are given at the bottom of the plot. Bubbles were jittered to better separate individual beetle data points.

In the first set of tests with the drum off, female beetles turned left a total of 1,403 times and right 1,468 times, indicating there was not a population left:right bias. Figure 4 depicts the proportion of left turns for all beetles. While there was not a population turning bias, individual beetles often had a left:right bias, 41% percent of individual females tested with the drum turned off deviated significantly from a 50∶50 left:right ratio. At α = 0.05, one expects 5%. With the drum turned on, 65% of the individual female tests were significant. There is a statistically significant difference between these two ratios (for drum-on 30 significant, 16 not significant; for drum-off 21 significant, 30 not significant, X^2^ = 4.68, p = 0.03, chi-squared test, 1 d.f.). There were also a greater number of left turns than right turns with the drum on (2,178 versus 1,232; Figure 4).

Although fewer males were tested, there were similar numbers of total left:right turns in the drum-off condition (659∶652 drum-on, 1,086∶1,089, drum-off: note that more males were tested in the drum-off condition). Like females, there was no population level left:right bias. There were strong individual left:right turning biases (68% differed significantly from the 50∶50 ratio with the drum off, 40% with the drum on). For males, there was not a statistical difference between the two conditions (for drum-on six significant, nine not significant; for drum-off 17 significant, eight not significant, X^2^ = 1.97, p = 0.16, chi-squared test, 1 d.f.). While numbers of males tested were smaller than for females, resulting in lower power, the trend was also in the opposite direction (fewer males showing a left:right bias in the drum-on condition).

**Figure 5:**
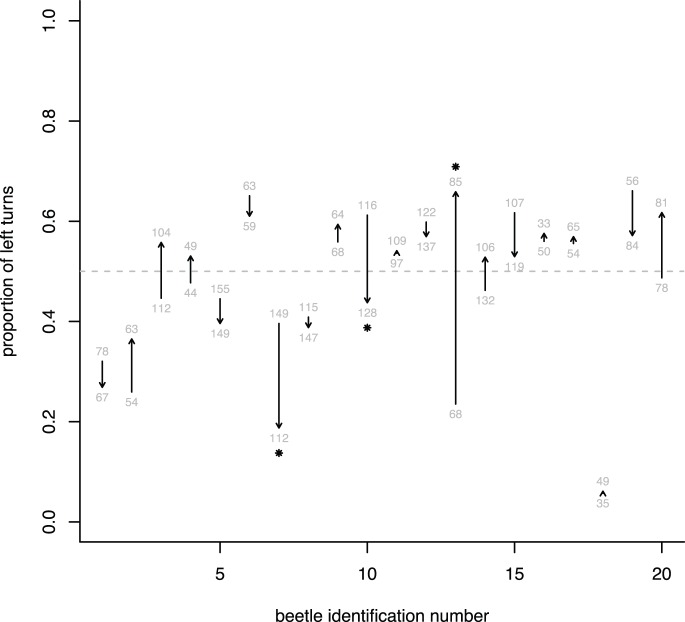
Proportion of left turns for male coffee berry borers from the drum-off condition to the drum-on condition. An arrow is drawn from the proportion of left turns from the drum-off condition to the drum-on condition for each male beetle. An asterisk at the arrowhead denotes that the distribution of turns differed for that male between the two drum conditions. The x-axis gives the beetle identification number (20 beetles were tested). Numbers (in gray) give the total number of turns, two for each beetle (drum-on and drum-off).

Since males overall did not exhibit a differential response when the drum was turned on, we decided to use the more sensitive paired test design, where we compared left:right turns with the drum off to left:right turns with the drum on for individuals. For these 20 paired tests, three showed a significantly different distribution of left:right turns under the two drum conditions (one significantly increased the proportion of left turns when the drum was on, in two they were significantly decreased; Figure 5). Thus, these paired tests indicate that males did not turn more often to the left when the drum was rotating.

In summary, individuals of both sexes often had a turn direction preference. Males showed no indication of perceiving the rotating drum in either the first or second set of tests, females showed a detectable response in the first set of tests.

## Discussion

Other bark beetles have been reported to have a reduced number of facets. Bright [10] reported on reduced female eyes in a cavernicolous population of *Coccotrypes dactyliperda* Fabricius (Table 1). Non-cavernicolous *C. dactyliperda* have a broad host range and can reproduce in fruits and seeds. Males do not exit their host [3] and have “poorly developed eyes” and “may be almost blind” [10]. Similarly, males of *Coccotrypes gedeanus* (Eggers) (reported as *Poecilips gedeanus* (Eggers)), which breed in fruits and seeds, never leave their hosts plants and “the eyes are so poorly developed that it is probably quite blind” [3]. Males in 14 out of 51 *Hypothenemus* species in South America are reported to have reduced eyes when compared to females [19]. Unfortunately, details on their biology are scant although most of them breed in stems or branches of tropical plants. Chu *et al.* [8] and Chu and Norris [9] reported a lower number of facets in male vs female *X. ferrugineus*, equivalent to 3–4 times lower (Table 1).

It could be argued that male coffee berry borers, which spend their entire life cycle inside a coffee berry, do not have a need for visual acuity, as do females, which do exit the berry and have to locate berries in which to oviposit.

This hypothesis is confirmed by the optomotor response results, which indicate that females do respond to motion, in contrast to males, who did not respond under the conditions tested. One would expect insects to be able to “see” a grating with 18° wide stripes if the interommatidial angles are less than the stripe width, i.e. Δ*Φ* is <18°. Therefore, the females, with Δ*Φ*∼13.6°, give a response that is close to what would be expected. However, the males, in which Δ*Φ*∼35.5°, would need a grating with stripes twice as wide to be able to see them. Thus we cannot rule out the possibility of males having some vision, but the resolution would be so bad as to be practically useless.

The biology of male coffee berry borers is analogous to Darwin’s description of “animals inhabiting dark caverns” whereby in the case of disuse of the eyes, it appears that “selection would continue slowly to reduce the organ, until it was rendered harmless and rudimentary” [1]. An example is blind cave fish, which lose their eyes in a million years or less because they have no use for them [20]. The reduced number of facets in coffee berry borer males appears to be an example of evolutionary adaptation in response to environmental conditions. This is supported by the S.D. values, which were similar for males and females despite the differences in the number of facets. A trait under stabilizing selection would tend to have low variability compared to the mean [21], [22]. A measure of this is the coefficient of variation (S.D./mean). For females, it is only 3.0%, while for males it is 21.5%, seven times as large. This suggests that the number of facets is under selective pressure for females, but not for males (because they do not use their eyes).

For 27 species of hymenopterans of many sizes, the ommatidial diameter varies as the square root of eye height, in keeping with the idea that all species are maximizing resolution within the space resources available [23]. In addition, the eyes retain a regular structure with the ommatidia tightly packed. For male coffee berry borers this is not happening. The male facets are loosely and rather randomly distributed with large spaces between them, and are the same size as in the female. This all indicates that this is not a properly functioning compound eye in which acuity is being maximized in an eye of smaller size. Presumably this is because selection pressure for optimized vision has been relaxed. It can be argued that eyes are expensive to run and maintain [24] and if not needed, will atrophy, which is what seems to be happening with male coffee berry borers.
